# The complex interplay between aging and cancer

**DOI:** 10.1038/s43587-025-00827-z

**Published:** 2025-03-04

**Authors:** Lucrezia A. Trastus, Fabrizio d’Adda di Fagagna

**Affiliations:** 1 IFOM ETS - The AIRC Institute of Molecular Oncology, Milan, Italy; 2 Istituto di Genetica Molecolare, Consiglio Nazionale delle Ricerche (IGM-CNR), Pavia, Italy

## Abstract

Cancer is an age-related disease, but the interplay between cancer and aging is complex and their shared molecular drivers deeply intertwine. This review provides an overview of how different biological pathways impact cancer and aging, leveraging the evidence mainly derived from animal studies. We discuss how genome maintenance and accumulation of DNA mutations affect tumorigenesis and tissue homeostasis during aging. We describe how age-related telomere dysfunction and cellular senescence provide a complex drive to tumor development, involving genomic instability and inflammation. We examine how an aged immune system and chronic inflammation shape tumor response, fueling DNA damage and cellular senescence. Finally, since animal models are important to untangle the relative contributions of these aging-modulated pathways in cancer and test interventions, we discuss some of the limitations of physiological and accelerated aging models, aiming to improve experimental designs and enhance translatability impact.

In 2022, almost 20 million cancer diagnoses and 10 million related deaths were reported globally^1^. Approximately one in five individuals develops cancer in a lifetime^1^, making it the second leading cause of death worldwide^2,3^. Malignancies can potentially arise throughout the lifespan, however half of all tumors are identified in people older than 70 years^4^. The surprising observation that cancer incidence seems to decline after age 80^2,4^ has been challenged by autopsy data suggesting it may be due to reduced diagnostic intensity in the older population at least for some cancer types^5^. Therefore, with overall tumor incidence increasing with age and aging being the greatest risk factor for cancer, cancer is generally considered an age-related disease.

Several mechanisms link aging to cancer. Over time, cells of the organism accumulate genomic, epigenomic, and proteomic damage, due both to intrinsic repair limitations and to a decrease in cell maintenance mechanisms^6^. The progressive buildup of dysfunctioning cell components contributes to the functional decline that characterizes and causes aging. If such dysfunctional cells are not effectively eliminated, this may favor the emergence and expansion of transformed cells. From the evolutionary point of view, diseases occurring after reproductive age are under a weaker selective pressure allowing the observed increase in cancer incidence in older age.

The observation that many, although not all, accelerated aging conditions are associated with increased cancer incidence^7–9^ further supports the notion of cancer as an age-related disease. Vice versa, interventions that increase lifespan and healthspan, including diet, physical activity, or pharmacological treatments, frequently reduce tumorigenesis^10,11^. Consistent with that, animal species with long lifespans such as bat^12^, naked mole-rat^13,14^, and whale^15^ generally display low cancer incidence.

Although the links between cancer and aging have been studied for long time, their molecular drivers are still heatedly discussed. Aging and cancer are multifaceted phenomena controlled by several intertwined factors^6,16^. Some are shared, such as genomic instability and chronic inflammation, others have antagonistic or ambivalent roles, such as cellular senescence and telomere attrition^17^. Thus, the relative contribution of distinct aging pathways in the process of cancer development is complex and context-dependent.

In the present review, we analyze the role of genomic and epigenomic instability, telomere maintenance, cellular senescence, and immune function in aging and cancer, also discussing some potential therapeutic strategies that exploit these mechanisms. To dissect the causal links between these aging pathways and cancer development, we leverage results obtained in animal models, highlighting also some current limitations of their use to study cancer initiation, progression, and response to therapy. Here, we chose not to address other important aging pathways and aspects of cancer biology, such as metastatization and therapy resistance.

## The role of mutations and clonal expansion in aging and cancer

Cancer is generally considered a genetic disease that develops over time through a set of somatic mutations, progressively conferring proliferative advantage to cells. Therefore, organisms that live long enough will potentially develop tumors, due to the inevitable accumulation of DNA replication errors and mistakes in DNA damage repair. In this sense, the passage of time, measured by chronological age, makes an important contribution to cancer risk.

Theodor Boveri was one of the first scientists who foresaw that one of the causes of transformation was genomic instability^18^, a hallmark shared by cancer and aging^17^. However, mutation burdens in tumors can vary widely, with pediatric tumors usually having few mutations and tumors induced by cigarette smoke or UV rays having many^19^. Some tumors display clusters of mutations arising from single catastrophic events occurring early in the transformation process, such as chromothripsis caused by telomere crisis^20,21^, whereas others are associated with chromosomal aneuploidy without known driver mutations^20^. Illuminating in this regard is the observation that intestine sections of the colon and ileum bear similar mutational burdens, but the former displays higher cancer incidence^22^. Despite the widespread notion of higher mutational load in tumors compared to corresponding normal tissues^22,23^, this heterogeneity indicates that mutation abundance may not be the only factor that determines cancer development. Technical limitations may also contribute to mask differences between normal and cancerous cells, since chromosomal instability and aneuploidy are more difficult to detect with current sequencing technologies^24,25^. Indeed, one disparity may lie in the extent of large structural variations that heavily compromise cellular functionality, while small lesions might be more tolerated in normal cells^26^. Both mutations and genome instability may progressively foster tumorigenesis by offering a replicative advantage and favoring the generation of additional aberrations.

Mutations may contribute to age-related phenotypes too, as recently it was observed that mutation accumulation is not restricted to cancer cells and the number of base substitutions and structural variations increases with age also in normal tissues^22,27–29^. Also mitochondrial genome mutations accumulate in aged and cancer tissues, possibly contributing to both processes^6,30^. Noteworthy, DNA mutations, even when not conferring a proliferative advantage, may alter gene expression and therefore cell functionality. In addition, cumulative DNA damage may result in transcriptional noise^31^, further compromising gene regulatory networks robustness and tissue homeostasis when enough mutated cells accumulate^32^.

An unexpected striking similarity to cancer in apparently normal aged issues is the reported expansion of mutant cells with competitive advantage, as previously observed in the skin^33^. The best characterized example is probably clonal hematopoiesis of indeterminate potential (CHIP), observed in up to 10-20% of the population over 70 years old^34^. CHIP is a non-malignant condition where a substantial proportion of blood cells originate from a single hematopoietic stem or progenitor cell bearing mutations that provide a selective growth advantage, predominantly in genes involved in DNA damage and epigenetic regulation^34,35^. CHIP is considered a pre-malignant state, associated with up to a 10-fold higher risk of developing hematopoietic malignancies, cardiovascular diseases, and all-cause mortality^36^. A more recent observation was made in the aged healthy esophageal epithelium where both the number of mutations per cell increases and cellular clones enlarge and multiply, ending up constituting a substantial fraction of the organ^37,38^. More broadly, analyses of single-nucleotide variants and small insertions and deletions revealed that in individuals aged 60 years or older, the proportion of cells harboring oncogenic mutations reaches over 50% in multiple tissues^24^. Interestingly, some tissues exhibited the highest prevalence of cells bearing cancer-associated mutations at young age, like colon before 30 years^24^. This spread of *de novo* mutations generates tissue heterogeneity and genome mosaicism, which may have a causal role in the aging process through the loss of tissue homeostasis^32^.

The observation that aging seems associated with a linear increase in DNA mutations^39^ does not align with the evidence that some types of cancer show an exponential increase of probability of occurring with increasing age^19^. This may be explained by cell-intrinsic mechanisms, such as clonal expansion and progressive genome instability of aberrant cells, that increase the probability of transformation^22^. However, although the acquisition and expansion of driver mutations are common in healthy tissues, only a small fraction of all clones becomes overtly malignant^22^. The reasons for that are not clear and, paradoxically, clonal proliferation can have a tumor suppressive role, as shown by the observation that *Notch1*-mutant clones in normal esophagus actually eradicate early tumors^40,41^. In addition, age-related cell-extrinsic events, such as the senescent microenvironment that will be discussed in the following sections, may further contribute.

In conclusion, mutation numbers rise with age accompanied by the clonal expansion of the cells with replicative advantage. These two processes can promote aging through transcriptional dysregulation and loss of tissue homeostasis, but they can also be initial steps toward tumorigenesis. To counteract these undesirable possibilities, cells evolved DNA repair strategies capable of restoring the normal base-pair sequence.

## The role of DNA repair in aging and cancer

DNA damage consists of physical alterations of the DNA structure. Importantly however, DNA damage is not inherently mutagenic, rather, DNA mutations, which are changes in the genetic sequence, arise when DNA repair is inaccurate. Therefore, DNA damage is inconsequential when faithfully repairable, and thus when the genetic and epigenetic information is restored, instead, mutations are irreversible and can only be eliminated by cell death or constrained by cellular senescence, a state of permanent growth arrest. For these reasons, an efficient and faithful DNA repair process may retard mutation accumulation, and consequently aging and cancer occurrence.

Genetic defects in DNA repair typically cause accelerated aging syndromes often associated with cancer susceptibility and inflammation^7^. Accordingly, human centenarians have been reported to show increased DNA repair capacity^8,42^. Conversely, humans and animal models treated with genotoxic treatments like radiotherapy or chemotherapy show signs of accelerated aging^8^. Interestingly, the ability to repair DNA damage induced by bleomycin, a chemotherapeutic agent, or ionizing radiation correlates with the lifespan of different rodent species^43,44^. In line with these results, the overall mutational rate per year across mammalian species is inversely correlated with their lifespan^13^. Remarkably, the observation that mammals with different natural lifespans at the end of their life have similar mutation numbers suggests that the somatic mutation burden is connected to aging and has an upper limit. How mutation abundance may be evolutionarily constrained is unclear. One possibility is that organisms become dysfunctional above a threshold number of mutations or mutated cells, which may become incompatible with organized life. Thus, long-lived species may have evolved more efficient strategies to reduce the mutation load by repairing DNA more accurately^44^ and/or by eliminating or confining mutated cells^45^. Consequently, different mutation rates in time of humans and laboratory mice should be considered when modeling cancer and aging^13^.

Mutation accumulation is further exacerbated by the reported decline in DNA repair efficiency and fidelity with aging^8,46^; however, notably, the mutation rate is constant throughout the lifespan^39^. This paradox may be explained by a potential negative selection of the most altered cells through apoptosis or clearance by the immune system. Alternatively, the general decrease in proliferation rates in aged tissues may reduce the chances for replication errors, compensating for the reduced ability to faithfully repair DNA. Differential repair efficiency according to the replicative capacity may also explain why the rates of small lesions per year in post-mitotic cells, such as neurons, are comparable to those in proliferating tissues^39^. Even differentiated cells in blood and colon have a mutational load similar to the corresponding stem and progenitor cells at the same age^39^. Thus, the accumulation of mutations appears time-dependent rather than division-dependent, although errors arising during stem cell divisions seem to be a main driver for cancer development, possibly explaining its different incidence in different tissues^47,48^. These observations indicate that much remains to be discovered about the mechanisms of generation and accumulation of mutations and their impact.

In sum, a large body of evidence supports an association between improper DNA repair and aging, which also predisposes to cancer by facilitating mutation accumulation. Deficiencies in different pathways of DNA repair are related to a higher risk for specific tumors, for instance, mutations in the components of homology-directed repair *BRCA1* or *BRCA2* underlie forms of breast and ovarian cancer mainly, while dysfunctional non-homologous DNA end joining is connected to blood cancers^42,49^. Noteworthy however, not all genetic DNA repair defects associate with increased cancer risk^8^. A good example is the nucleotide excision repair pathway, in which defects in the global genomic branch predispose to cancer, while defects in the transcription-coupled branch lead to premature aging^50^. Also other DNA repair defects, including Lynch syndrome that is caused by defects in mismatch repair^42^, are associated with increased cancer incidence without obvious aging phenotypes. The reasons why cancer and premature aging are uncoupled in these syndromes remain unclear and genetically modified animal models can help clarify this interplay as animals with defects in DNA repair can develop tumors due to mutation accumulation and display features of accelerated aging^7,8^, noteworthy however pathology severity is usually milder in mice compared to humans^49^.

Besides mutation accumulation, DNA damage has additional consequences that may play a causal role in aging and cancer, for instance by inducing epigenomic changes.

## The role of epigenomic changes in aging and cancer

DNA damage induces reversible changes in the epigenetic marks surrounding the lesion by causing the redistribution of chromatin remodelers^7,42^, which however sometimes can also persist. For this and other reasons, aging is accompanied by the accumulation of epigenomic and chromatin changes, such as alterations in DNA methylation patterns, post-translational modification of histones, chromatin remodeling, and function of non-coding RNAs^6,51,52^. This altered epigenomic landscape has been proposed to drive aging and age-associated conditions^6,7^, including immune aging^53,54^, by altering gene expression and causing transcriptional noise^6,28,32^. Functional epigenetic marks are progressively lost during aging, contributing to the loss of cell identity and potentially leading to an increased risk of carcinogenesis. Several computational “epigenetic clocks” exploit the changes in DNA methylation patterns to predict chronological age, risk for age-associated diseases, and mortality^55,56^. Interestingly, DNA methylation drift is associated with age acceleration in mice with progeroid diseases, especially those with defects in DNA repair^57^, while its association with telomere dysfunction remains unexplored. In addition, members of the SIRT family of protein deacetylases have been shown to be involved in the modulation of age-related phenotypes^6,7^ and organismal lifespan^44^, although it is unclear whether these epigenetic mechanisms influence aging *per se* or by modulating DNA repair or other signaling pathways. As an example, SIRT6 silences the expression of retrotransposable elements such as LINE-1, thus SIRT6 relocalization in the genome of aged or senescent cells results in epigenetic derepression and LINE-1 transcription, with consequent genome instability and inflammation^6,7^. Accordingly, inhibition of retrotransposition improved lifespan and phenotypes in physiologically aged or progeroid mouse models^6^. It has recently been proposed that faithfully repaired DNA double-strand breaks (DSBs) may induce permanent changes in surrounding chromatin thus promoting aging at cellular and systemic levels, suggesting that the erosion of the epigenomic landscape due to DNA damage can intrinsically contribute to aging^58^. More in general, the observation that epigenetic reprogramming through expression of the OSK(M) Yamanaka factors can reverse age-related phenotypes and rejuvenate epigenetic clocks^6,59^ supports the notion that epigenomic changes play a causal role in the aging process.

Altered epigenetic modifications are shared in aged and cancer cells^17^ and may promote tumorigenesis. Indeed, gene expression can be distorted not only by genomic mutations but also by “epimutations”, which are alterations in chromatin structure and epigenetic marks despite unaltered DNA sequence. Through epimutations, cancer cells can increase their fitness and adaptation to stress or acquire new properties such as invasive ability, phenotypic plasticity, or telomere elongation^16,60^. By acting on DNA methylation, cells can modify the expression of oncogenes or tumor suppressor genes, or silence DNA damage and repair genes increasing genomic instability^61^.

Nevertheless, evaluating the causal role of epimutations in aging and cancer can be challenging because it requires integrating multi-omics techniques to detect them and determine their impact on gene expression, and specifically reverting them to demonstrate causality.

**Figure 1:**
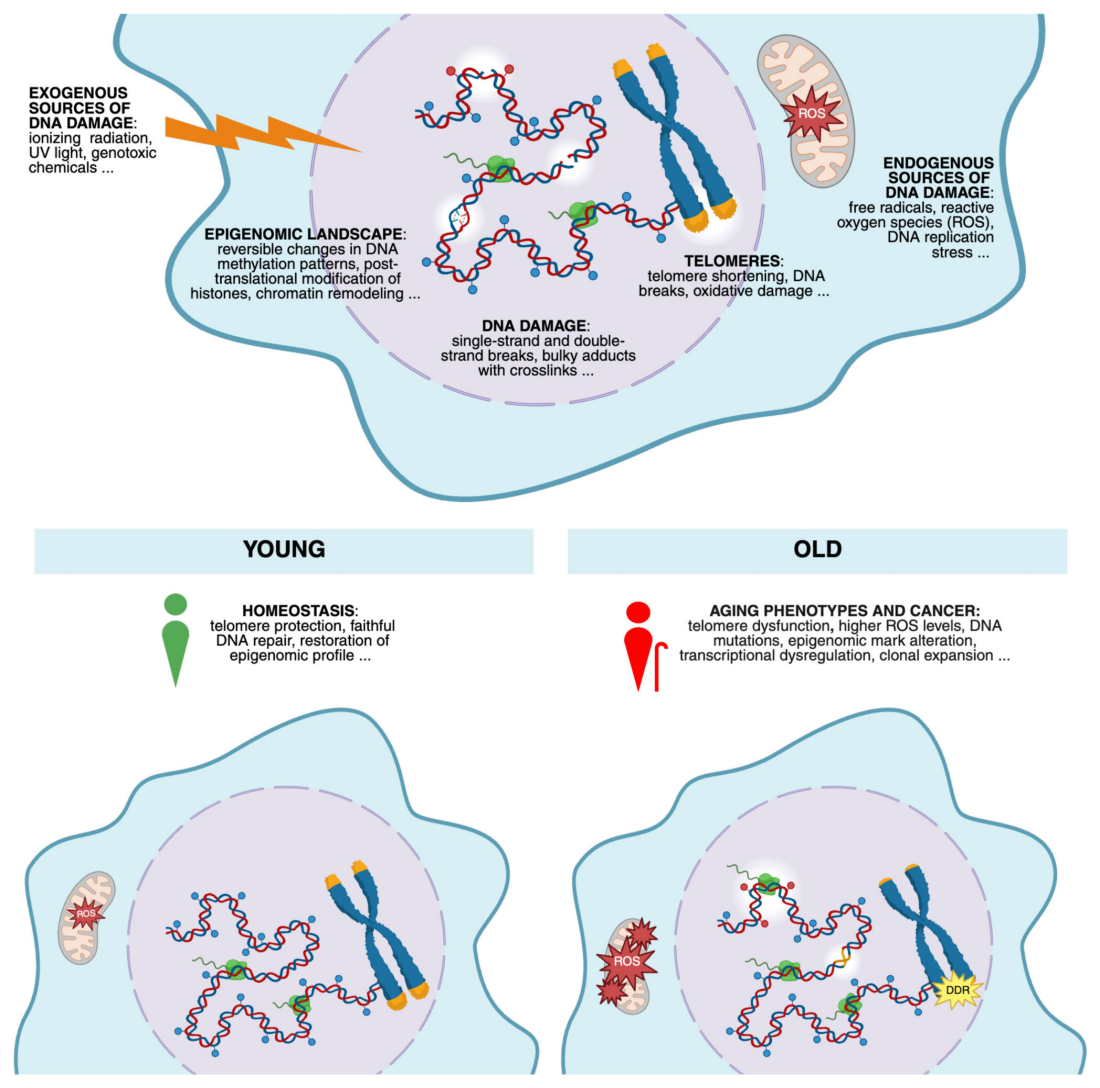
The role of DNA damage in aging and cancer. Throughout life, DNA integrity is threatened by exogenous agents, such as ionizing radiation or UV light, or endogenous processes, including oxidative or replication stress. The resulting DNA damage may range from nucleotide modifications to single and double-strand breaks and leads to transient epigenomic changes^7,27,28,32^. Moreover, telomeres progressively get short and damaged in time^63^, especially due to oxidative stress^60,64,73^. In early age, homeostasis is maintained because DNA damage generation is effectively counteracted by the ability to faithfully re-establish the original DNA sequence and epigenetic information, and to maintain telomere integrity. During aging, instead, ROS load increases^6^, DNA replication errors accumulate, and DNA repair ability declines^8,46^, thus lesions can be converted into mutations and epigenetic marks can become permanent^6,7^, resulting in transcriptional dysregulation^6,28,32^. Mutated cells that acquired a proliferative advantage expand, forming cellular clones that may become a significant proportion in the tissues^24,32^. Finally, due to their irreparability^66,67^ and insufficiency of telomere maintenance mechanisms in differentiated cells^63^, the number of damaged and critically short telomeres increases in time^66,171^, leading to persistent DDR activation and cell death or cellular senescence establishment. Collectively, the consequences of DNA damage in the older population can cause age-related tissue dysfunction and tumorigenesis.

The genetic and epigenetic processes discussed so far contribute to aging and cancer in an intricate manner (Figure 1). Progressively inefficient and unfaithful repair of the DNA damage generated over time leads to the expansion of genetic and epigenetic mutations. Mutant cell clones expand with time and fuel tumorigenesis and age-related phenotypes. While the causal role of mutations in cancer has been proven, their contribution and the one of epimutations to aging have not been demonstrated yet, also because of the difficulty in unraveling the specific impact of mutations, since DNA damage is intrinsically required for their generation. An additional complication is that DNA lesions by themselves can contribute to cancer and aging by resisting DNA repair. In agreement with the potential role of unrepaired DNA damage in aging, markers of DNA damage such as oxidized guanine (8-oxodG) and the phosphorylated form of histone H2AX (γH2AX) are more frequent in cells from several tissues of old mice and humans^8^. Unfortunately, direct detection of DNA damage is technically more challenging compared to mutations and further research is needed to map and quantify *in vivo* DNA lesions accumulation with age. Known examples of genomic sites in which DNA repair is impaired are the telomeres.

## The role of telomere maintenance and dysfunction in aging and cancer

Genomic DSBs normally trigger the activation of the DNA damage response (DDR), a set of coordinated events that signals the presence of DNA damage resulting in the enforcement of cell-cycle checkpoints and DNA repair efforts. DDR activation remains sustained until DNA damage is resolved, but, if repair is not achieved, chronic DDR signaling promotes cellular senescence or cell death. Telomeres are the ends of linear chromosomes, hence they structurally resemble DSBs. However, the telomere-bound shelterin protein complex inhibits DDR signaling and repair, thus preventing cell death/cellular senescence and telomeric fusions, respectively^62^. In proliferating cells, telomeres progressively shorten because of the end replication problem, namely the inability of the standard DNA replication machinery to fully replicate linear DNA molecules, and because of nucleolytic activities^63^. Embryonic, germinal, and stem cells generally counteract telomere attrition by expressing telomerase, a ribonucleoprotein that reverse transcribes its associated telomeric RNA template to extend telomeres^64^. Instead, in somatic human cells, telomere shortening progressively leads to a reduction of binding sites for shelterin complexes eventually becoming unable to inhibit DDR signaling while still able to prevent chromosomal fusions^63^. This fuels persistent DDR signaling leading to the establishment of cellular senescence or cell death^65^. This mechanism limits the number of cell divisions that normal somatic cells can achieve^50^, primarily dictated by the length of the shortest telomere(s). Telomeric damage is also crucial in non-proliferating cells, indeed, shelterin proteins, by fulfilling their evolutionary-selected role of preventing telomeric fusions, actively inhibit the repair of DSBs occurring within telomeric repeats. Thus, if telomeric DSBs are generated, they cannot be repaired and fuel persistent DDR signaling, leading to a cellular senescence-like state or apoptosis^66,67^. Therefore, the loss of telomere integrity, including critically short^65^ and long but damaged telomeres^65–69^, promotes cellular senescence in dividing and non-dividing cells. Hence, telomere dysfunction, rather than telomere shortening only, is causally associated with age-related diseases, as extensively reviewed in^63^. Indeed, although telomere length anticorrelates with age and is considered an aging biomarker, it does not inform on the contribution of long but damaged telomeres. Lifespan of different species is indeed correlated with the accumulation of critically short telomeres (Box 1), determined by the combination of inherited length and shortening rates. Thus, since telomere shortening is not linear in time^70^ and has different rates in different tissues^71^, it might be useful as a “replicometer”^72^, while the accumulation of dysfunctional and DDR-signaling telomeres may be a better biomarker for aging and age-related diseases^69,73^. To adopt this aging biomarker, loss of telomere integrity can be quantified by assessing the localization of DDR factors at telomeres, or indirectly by detecting the abundance of telomeric RNAs, which have been reported to be transcriptionally induced at damaged telomeres^74–76^.

Cancer cells are under selective pressure to gain unlimited proliferation potential by acquiring telomere maintenance mechanisms^77^. Two main telomere elongation strategies are known: the most common is telomerase reactivation, while the recombination-based alternative lengthening of telomeres (ALT) has been estimated in 5-15% of tumors^78–80^. Rarely, if telomeres are sufficiently long at the time of transformation, cancer cells may not need to adopt telomere maintenance mechanisms^81–84^, an observation that challenges the widespread notion of replicative immortality as a cancer hallmark^16,85,86^.

Mutations in telomere maintenance-associated genes can cause telomere biology disorders, associated with too short or too long telomeres. While patients with short telomeres display accelerated aging and age-related conditions, such as pulmonary fibrosis and bone marrow failure, patients with long telomeres are predisposed to cancer, in particular melanoma and chronic lymphocytic leukemia^60,70,71,87^. This is consistent with the hypothesis that telomere erosion may have been evolutionarily selected for its advantage early in life by limiting cell proliferation and thus malignant progression, with the unselected drawback of promoting aging later in life. However, telomere shortening is not tumor suppressive in all settings, because cells with critically short telomeres that escape cellular senescence by mutating checkpoint genes will proliferate and the consequent telomere attrition can lead to further loss of sheltering binding allowing chromosomal fusion and rampant genome instability^21,77^. Indeed, cancer development and progression can be accompanied by telomere-driven genomic abnormalities^64,77^.

Animal models have been adopted to study the impact of telomere length on human pathologies, yet important caveats apply. Laboratory mice display longer telomeres, faster shortening rate, and express telomerase more widely among differentiated cells compared to humans^64,88^. Other animals like zebrafish (*Danio rerio*) might be better models in terms of telomere biology because of their human-like features, including telomere size and tighter telomerase expression control^89^.

One of the most common strategies to dissect the impact of telomere biology on aging and cancer is to modulate telomere length, thus scientists adopted telomerase knockout mice, in which telomeres progressively shorten with each generation^90^. However, a limitation of this model consists in its genetic inactivation in all the cells, including germinal, stem, and progenitor cells, which in humans express telomerase^64^. In addition, the inactivation of the protein, but not the RNA, component of the telomerase complex impairs also its non-catalytic functions unrelated to telomere biology^64,91^. Progeroid phenotypes and lifespan reduction become apparent upon telomerase inactivation only when mice reach late generations^92^, and telomeres engage DDR. Accordingly, telomerase reactivation in late generation mice suppresses this signaling and the associated cellular checkpoints, alleviating the functional decline of several tissues^93^. In this regard, zebrafish, whose telomere length is comparable to humans, display pathological phenotypes at the first generation upon telomerase homozygous inactivation^89^, similar to humans with heterozygous telomerase mutation^70^.

Regarding cancer and reflecting the context-dependent role of telomeres, also telomerase may have both tumor-promoting and tumor-suppressive functions. In line with the accelerated aging phenotype, late generations telomerase knockout mouse and zebrafish show increased spontaneous cancer incidence, likely promoted by increased genomic instability and inflammation caused by telomere dysfunction^92,94^. Interestingly, p53-deficient telomerase knockout mice show a humanized tumor spectrum, while typically murine tumors have different origins and minimal structural aberrations compared to human^64^. However, also mice that overexpress telomerase, but not mice with hyper-long telomeres without genetic manipulation^95^, are more prone to tumor formation, but show longer lifespan if they survive cancer^96^. Indeed, telomerase activity is upregulated in mouse tumorigenesis^96^, indicating positive pressure for telomerase functions, perhaps including the telomere-independent angiogenesis stimulation through increased VEGF expression^64,91^. Accordingly, several reports show a protective role of telomerase inactivation in different cancer settings^97–99^, and for this reason, telomerase inhibitors are being tested for anti-cancer treatments^60,100^. Reconciling these results, the role of telomeres and telomerase on tumorigenesis depends on the context: telomere dysfunction constrains cancer cell proliferation through DDR activation and senescence, but bypassing telomere-driven senescence through inactivation of checkpoint genes allows genome instability to fuel tumorigenesis. In line with this, telomerase overexpression by adeno-associated viruses does not accelerate tumor onset or progression in cancer-prone mice^101^, but upon telomere dysfunction, telomerase reactivation causes more aggressive tumors^99^, likely by reducing telomeric DDR signaling and promoting malignant cell proliferation. Thus, the status of p53, a key node of DDR checkpoint enforcement, can dictate whether telomere dysfunction enhances or suppresses cancer development.

In conclusion, telomere shortening plays tumor suppressive functions by limiting cell proliferation, but it turns into a promoter of genomic instability and cellular transformation upon DDR signaling impairment, illustrating the complex interplay between mutations in checkpoint genes and telomere maintenance. In parallel, the accumulation of cells with dysfunctional telomeres is associated with age-related phenotypes and lifespan. These telomeric roles are mediated by cellular senescence, which acts as a bridge between DNA damage, aging and cancer.

## The role of cellular senescence in aging and cancer

**Figure 2:**
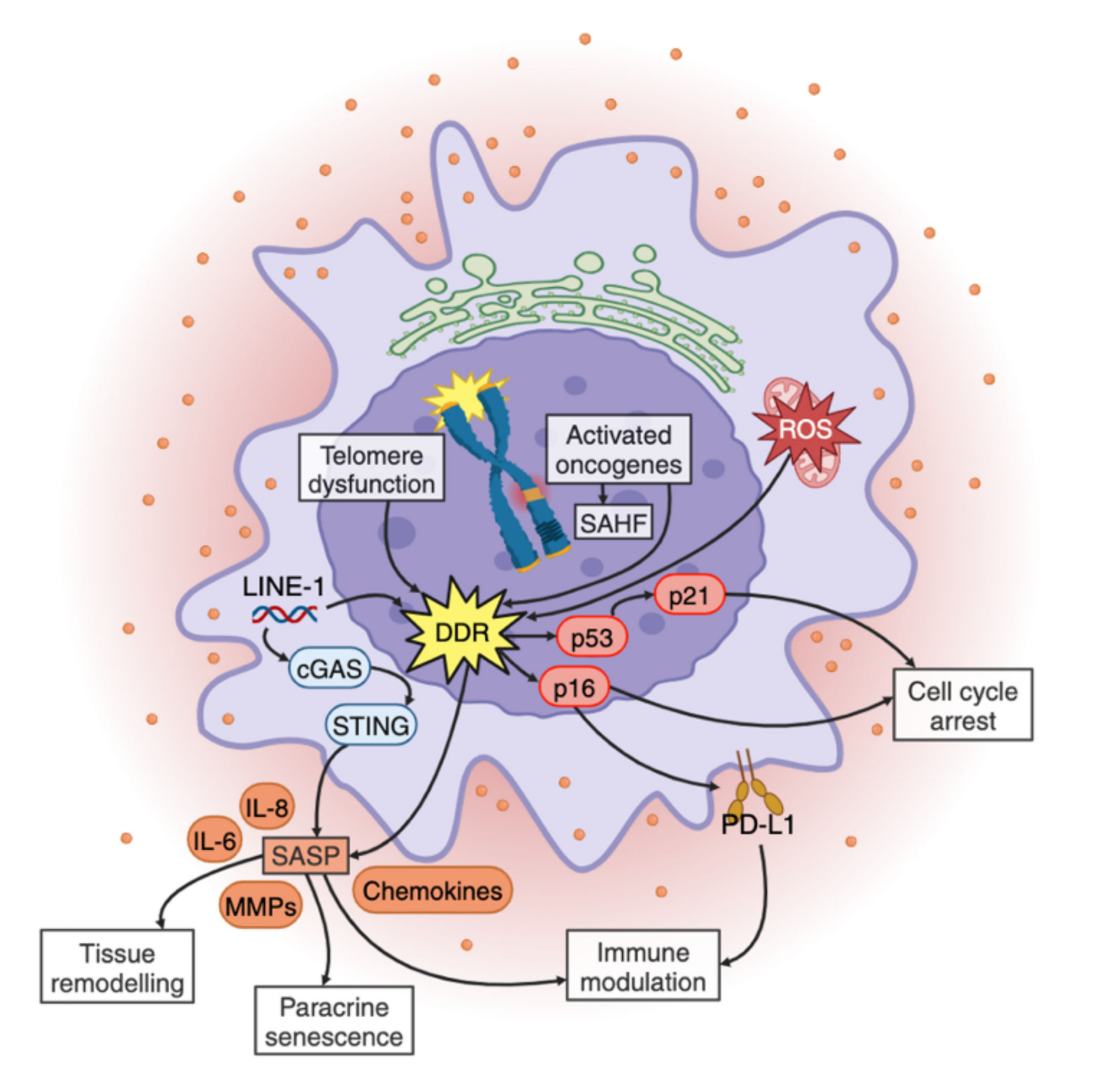
Molecular mechanisms and phenotypes of cellular senescence. Cellular senescence ensues upon different persistent stress events and encompasses a variety of molecular features, only some of which are depicted in this figure. Telomere dysfunction, increased reactive oxygen species (ROS) load, or oncogene activation may trigger a persistent DDR, which in turn leads to permanent cell cycle arrest mediated by CDK inhibitors like p21 and p16, and the senescence-associated secretory phenotype (SASP)^8,102^. The SASP involves the secretion of pro-inflammatory cytokines, such as IL-6 and IL-8, chemokines, and matrix metalloproteinases (MMPs), which remodel the microenvironment, spread paracrine senescence, and modulate the immune response^102,114,126^. In senescent cells, chromatin is reorganized and, in the case of oncogene activation, senescence-associated heterochromatic foci (SAHF) are formed, but their role in SASP is yet to be elucidated^102,103^. Additionally, p16-driven expression of programmed death-ligand 1 (PD-L1) and PD-L2 promotes an immunosuppressive environment and accumulation of senescent cells^116–118^. In addition, LINE-1 retrotransposon deregulation in senescent cells activates the DDR and cGAS-STING sensing pathway, further amplifying inflammation^6,7^.

Cellular senescence is a state of stable cell cycle arrest characterized by enlarged cell appearance, altered gene expression, apoptosis resistance, and a senescence-associated secretory phenotype (SASP) (Figure 2). The SASP is a transcriptionally and epigenetically-controlled^102,103^ program that promotes the secretion of pro-inflammatory cytokines, extracellular matrix proteases, and angiogenic factors. Cellular senescence was first observed by Hayflick and Moorhead in primary human fibroblasts upon multiple passages in culture^104^, therefore the “Hayflick limit” indicates the restricted capacity of normal cells to divide until they reach replicative senescence. Various types of cellular senescence can arise depending on the stress that triggers it, including oncogene activation, persistent DNA damage, and telomere dysfunction, all sharing chronic DDR activation, with some exceptions^8,102^. For this reason, cellular senescence acts as a tumor-suppressor mechanism to restrain the proliferation of oncogene-expressing and damaged cells. Interestingly, while the causality of oncogene-driven chronic DDR signaling in driving cellular senescence has been proved^105^, the contribution of mutations in genes unrelated to cancer has not yet been determined. This ambiguity persists in part because experimentally decoupling the role of DDR signaling from the one of mutation generation is challenging, given that both are consequent to a DNA damage event.

Along with DDR activation^66^, senescent cells^102,106–108^ and circulating SASP components^109^ accumulate with aging and more so in age-related diseases^63^. Despite their relatively low abundance, senescent cells impair tissue homeostasis due to their cell-intrinsic inability to proliferate and their cell-extrinsic SASP, impacting surrounding cells and eventually producing a systemic effect. Indeed, the secretory phenotype acts through autocrine and paracrine mechanisms^110–112^, respectively reinforcing and spreading senescence. SASP effect on the tissue microenvironment can be beneficial, recruiting immune cells to clear senescent cells^113^. However, with aging a threshold burden may be reached, beyond which senescent cells exceed the clearance capacity of immune cells^114^, which when becoming senescent themselves may become less able to remove damaged cells^115^. The expression of programmed death-ligand 1 and 2 (PD-L1 and PD-L2) by some senescent cells provides an additional mechanism to escape clearance and promote their accumulation^116–118^. Therefore, the exponential increase of senescent cells with aging fits a model that combines their linear accumulation due to senescence-inducing stimuli and a slowdown in their removal dependent on their abundance^119^.

Just like telomere dysfuntion, cellular senescence can be advantageous in early life but may cause detrimental age-related phenotypes later in life^63^. The causal role of cellular senescence in localized and systemic age-related phenotypes was demonstrated in animal models when few senescent cells injected in young or adult mice caused lung fibrosis, widespread physical dysfunction, and shortened lifespan^112,120,121^. Conversely, mouse models that allow genetic depletion of senescent cells, such as the INK-ATTAC^122^ and p16-3MR^123^, were fundamental to prove that senescent cell removal ameliorates age-related phenotypes and healthspan^102,114,124^. Accordingly, senotherapies such as senomorphics (that act mainly by inhibiting SASP) and senolytics (that kill senescent cells), including senolysis mediated by chimeric antigen receptor (CAR) T cells^125^, improved several age-related conditions in numerous *in vivo* studies^102,114,120^. Accordingly, cellular senescence has a causal association with several age-related pathologies, spanning from pulmonary fibrosis to neurodegenerative diseases^63^. Cellular senescence may cause age-related phenotypes particularly when occurring in stem cells, contributing to tissue dysfunction by the loss of stemness and regenerative capacity^102^. The paracrine systemic effect of senescent cell accumulation in aged tissues is the induction of a chronic, low-grade inflammatory state contributing to multiple age-related disorders and immune dysregulation^53,54^.

In cancer, cellular senescence exhibits both tumor-suppressive and tumor-promoting functions. Activation of oncogenes, best characterized with members of the *BRAF* and *RAS* families^126^, in normal cells can result in “oncogene-induced senescence” (OIS), a proliferative barrier best observed *in vivo* in skin naevi and reported in several other premalignant lesions^127–131^. OIS prevents tumor progression in a cell-intrinsic manner, indeed repressing heterochromatin-dependent cellular senescence in a mouse model of OIS fuels tumor development^128^. Accordingly, the heterochromatic senescence-associated heterochromatic foci (SAHF) are hallmarks of OIS retained upon transformation, and their perturbation by histone deacetylase (HDAC) inhibitors unleashes DDR activation, causing apoptosis in cancer cells and tumor regression *in vivo*^132^. In addition, SASP promotes immunosurveillance, thus reinforcing tumor suppression in a cell-extrinsic manner^110,126^. However, senescent cells that manage to escape cell cycle arrest by downregulating tumor suppressor pathways can transform into malignant cells^126^. Accordingly, senescent tumor cells are more abundant at the early stages of tumorigenesis, compared to advanced lesions both in mice and humans^108,127–131,133^.

Paradoxically, however, cellular senescence can also promote cancer. SASP stimulates cancer growth as demonstrated by the observation that endogenous irradiation-induced^134^ or exogenous injected^135–137^ senescent cells fuel tumor progression. Interestingly, this tumor growth advantage driven by co-injection with senescent cells is lost when employing athymic immunodeficient mice, highlighting the ability of senescent cells to steer the immune response^137^. SASP can promote cancer progression by reprogramming the tumor microenvironment to a state more permissive for the growth or invasion of malignant cells^134,135^, through inflammation^127^, immunosuppressive cell recruitment^137^, and promotion of angiogenesis^113,126^. Indeed, senescent cell removal can reduce the growth of tumors injected or induced in immunocompetent mice^127,134^ or delay their spontaneous formation^124^. Thus, cellular senescence enforces tumor suppression through cell-intrinsic (proliferative arrest via OIS) and cell-extrinsic (immune clearance via SASP) mechanisms, but it also stimulates tumor growth and aggressiveness by cell-extrinsic mechanisms (microenvironment remodeling via SASP).

Therapy-induced senescence (TIS) as a result of chemotherapy and radiotherapy has been reported to cause inflammation and decrease immune function^126,138^, thus limiting the beneficial effects of these cancer therapies and contributing to their side effects, promoting secondary malignancies and accelerated aging, also measured by epigenetic clocks^56,139^. In this regard, senotherapies may improve the efficacy and reduce adverse effects of cancer therapies^102,126,138^, as indicated by the ability of the senolytic agent navitoclax to induce cancer regression in mouse xenografts upon TIS^140–142^ and by senolytic CAR T cells to increase survival of a cancer-prone mouse model of TIS^143^. Overall, animal models have been crucial to demonstrate that the accumulation of senescent cells plays a causal role in aging and cancer and to successfully test senotherapies that are now undergoing clinical trials^11,114^.

In conclusion, cellular senescence can prevent transformation but must be restricted by immunosurveillance, which counteracts its paracrine spread and inflammation that otherwise can fuel tumorigenesis and age-related diseases. When the balance between clearance and senescence establishment is lost, tissue homeostasis is compromised and cancer favoured.

## The role of immune function and inflammation in aging and cancer

**Figure 3:**
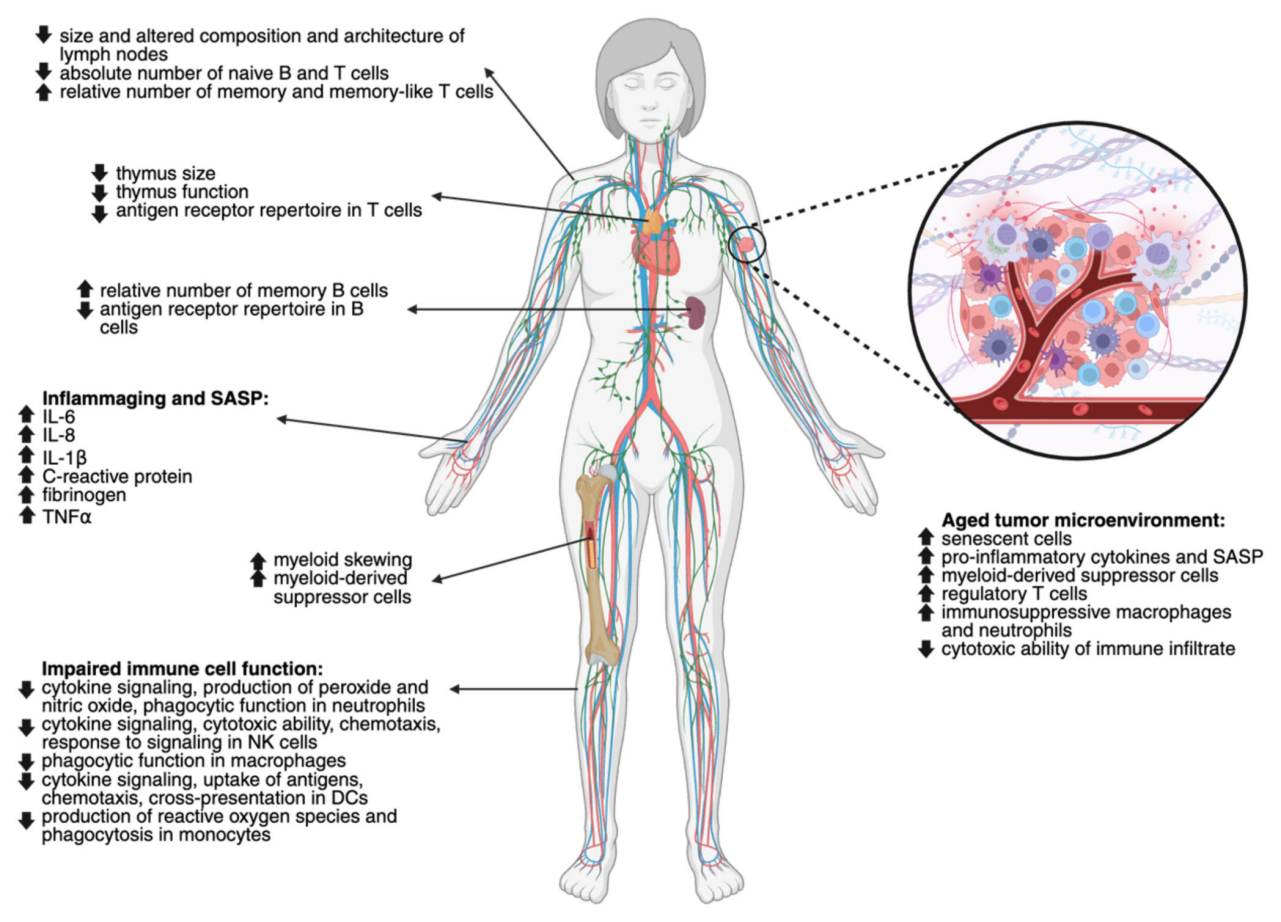
Immunosenescence phenotypes in aging and cancer Immunosenescence is the set of immune changes during aging that affects both the adaptive and innate immune functions and involves a reorganization of immune organs and an alteration of immune cells and circulating factors, determining age-related conditions and poor response to infections and vaccines^53^. These immune modifications encompass thymic involution^53,113^, changes in the composition and architecture of secondary lymphoid organs^144^, an increased differentiation potential to the myeloid lineage in the hematopoietic stem cell population (termed “myeloid skewing”)^102^, a reduction in the relative proportion of naïve lymphocytes^53,113,144^, and an increase in the relative proportion of memory lymphocytes^53,113,144^, however with a restricted antigen-receptor repertoire^53,113,144^. Neutrophils, macrophages, natural killer (NK), and dendritic cells (DCs) show altered cytokine secretion and weaker cytotoxic activity^54,113,144^. Finally, aging is associated with a low-grade persistent level of pro-inflammatory cytokines and chemokines in the serum, contributed by the SASP, including interleukin 6 (IL-6), IL-8, IL-1β, and tumor necrosis factor α (TNFα)^54,113,144^. The altered immune function during aging also impacts the composition of the aged tumor microenvironment which participates in tumor progression^53,54,113,148^: primary tumors are surrounded by senescent fibroblasts that contribute to maintaining an inflammatory microenvironment by their SASP, the immune infiltrate becomes rich in immunosuppressive myeloid-derived suppressor cells (MDSCs) and regulatory T cells (T_reg_), macrophages and neutrophils switch toward immunosuppressive states, and the cytotoxic ability of the infiltrated immune cells decrease.

During aging, several events lead to a decrease in the overall immune function, a phenomenon called “immunosenescence”^53,54,113,144,145^, which should not be confused with cellular senescence within the immune system, accompanied by a low-grade persistent level of pro-inflammatory molecules, often termed “inflammaging”, contributed by the SASP^53,54,144^ (Figure 3).

Although such immune changes have been proposed as an adaptation in older population to fight chronic infections with reduced energy consumption^146^, immunosenescence and inflammaging are causally associated with age-related diseases and malignancies^53,54^. A mouse model with hematopoietic-restricted cellular senescence caused by the inactivation of the DNA repair *Ercc1* gene demonstrated that a senescent immune system is sufficient to drive systemic senescence and widespread age-related phenotypes also in solid tissues^115^. A confirmation of the causal role of immunity in aging derives from the evidence that chronic low-grade inflammation in *nffib1* knockout mice induces premature aging by triggering telomere dysfunction-induced senescence^69^. Moreover, in humans reduced inflammation has been reported as the strongest predictor of healthy aging in centenarians^147^.

In physiological conditions, tumor development and progression are counteracted by the immune system, especially natural killer and T cells, and by acute inflammation^148^. This immune response is complex and highly context-dependent and relies on an orchestrated cellular cross-talk that may switch from anti-tumoral to pro-tumoral if the microenvironment is altered. Immunoediting is the process by which cancer development is dynamically shaped by the immune system through the elimination of the most immunogenic and mutated cancer cells and the emergence of immune-escaping variants^149^. Indeed, cancer cells need to evade immunosurveillance to spread^16^, and immune dysregulation and chronic inflammation facilitate this escape. Aged tissues^113^ are often associated with an immunosuppressive microenvironment that may foster tumorigenesis and metastatization, and tumors themselves^54,148^ can generate such microenvironments, also because the bone marrow can modify the hematopoietic output by sensing distant premalignant lesions^150^. Pollution-driven inflammation can promote cancer development through macrophage release of the cytokine interleukin-1β without enhanced mutagenesis. Indeed, exposure to particulate matter in mice harboring oncogenic mutations causes lung cancer in immunocompetent but not in immunodeficient animals^151^. This mechanism might be particularly relevant in older adults, where the expansion of mutated cells with proliferative advantage may provide a fertile ground for inflammation-driven tumorigenesis^19,24,34,38^, or where age-related chronic inflammation may unleash the expansion of mutant cells^35^. Interestingly, epidemiological evidence associates higher pro-inflammatory cytokine levels and inflammatory diseases with a higher risk of developing cancer^54,148^. In line with this, regular use of anti-inflammatory drugs has been linked to a lower risk of some cancer types, especially in the older population^152^.

An aged immune system may not only mount an inefficient anti-tumoral response but can even directly fuel tumor development and progression. Causative associations of immunosenescence and inflammaging with tumorigenesis derive from animal models. Upon injection of melanoma cells in immunologically-privileged sites, such as the eye, tumors grow slower in aged compared to young mice^153^. When injection is performed in non-privileged sites, such as the spleen, young mice develop fewer metastases compared to old mice or young mice that receive aged bone marrow transplantation (BMT), because of a less efficient response in the aged immune system^153^. Moreover, following conditional *H-ras* activation, aged but not young mice develop carcinoma, in association with the establishment of a pro-inflammatory immunosuppressive microenvironment in which senescent cells are less effectively removed by the immune system^154^. More strikingly, a recent study showed that an aged immune system, also when deriving from BMT, exacerbates emergency myelopoiesis in lung tumorigenesis, enhancing tumor growth^155^. BMT from young donors or pharmacological inhibition of interleukin-1α and β signaling pathway in an aged environment restore the anti-tumor response, delaying the progression of multiple cancer types in old mice. These examples illustrate how an aged immune system is more permissive to cancer growth and how useful are mouse models to study this. However, such models have limitations, since humans and mice show different kinetics and phenotypes of immunosenescence^145,156^ and laboratory animals are commonly kept in aseptic conditions^144^.

The fundamental aging pathways discussed in the previous sections are tightly interconnected in determining an impaired immune response to cancer. In general, senescent cells and SASP factors are associated with pro-tumorigenic processes, including chronic inflammation and immunosuppression^54,113,126^, but also senescent immune cells have a causal role in tumorigenesis. In fact, in lung tumors induced by *K-ras*, the same senescent macrophages that accumulate during aging also promote an immunosuppressive environment and thus cancer development, which can be reversed upon senescent cell or macrophage depletion^108,133^. Similarly, in telomerase-mutant zebrafish, anti-inflammatory drugs reduce the higher cancer incidence caused by telomere shortening-induced senescence^94^.

Telomere dysfunction too can be an aging driver for the immune system, ultimately leading to tumorigenesis. Besides the most obvious connection between telomeres and inflammation through cellular senescence and SASP, self-DNA generated by telomere dysfunction may trigger cGAS/STING-mediated inflammation^64,77^. Accordingly, animal models lacking telomerase display chronic inflammation and immune dysfunction^94,157,158^, and telomere biology disorder patients may have compromised hematopoietic and immune function^70,71,87^. Thus, damaged telomeres can cause chronic inflammation, but the other way around is also true. The *nffib* knockout mouse model demonstrated the existence of a positive feedback loop between inflammation and telomere dysfunction: genetically-induced chronic inflammation causes oxidative stress, the consequent DNA damage accumulates at telomeres causing persistent DDR activation that induces cellular senescence and SASP, which further intensifies the pro-inflammatory signaling in a self-perpetuating cycle^69^. This connection between telomeres and immunity can elicit tumor development as described in late-generation telomerase knockout mice, whose lymphopenia enhances the growth of immunogenic tumors^157^. Similarly, telomere biology disorder patients with short telomeres develop tumors because of defects in T-cell-mediated immunosurveillance, rather than genomic instability^157^. Likewise, DNA damage, mutations and epimutations can be drivers of the aging of the immune system, leading to compromised immunosurveillance, chronic inflammation, and ultimately tumorigenesis. For instance, premature aging syndromes caused by mutations in genes involved in DNA repair^7^ are associated with a chronic state of inflammation. Moreover, CHIP is associated with conditions involving dysregulated immune signaling, and inflammatory cytokines have been shown to drive the proliferation of hematopoietic mutated cells in a self-reinforcing loop^34,35^.

**Figure 4:**
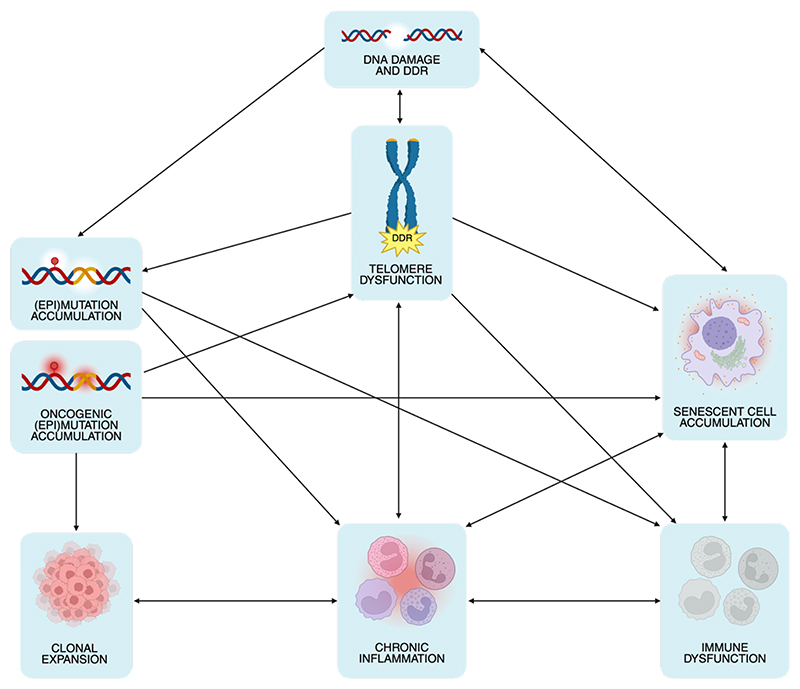
The complex interplay between aging and cancer. Several pathways are interconnected in causing age-related phenotypes and cancer. DNA damage triggers the DDR pathway, but DNA repair may be inaccurate, introducing genomic mutations and permanent epigenomic changes^7,27,28,32^. Cells expressing mutated oncogenes undergo OIS^102^, while the relationship between non-oncogenic mutation burden and cellular senescence remains unclear. Cells that acquire a proliferative advantage, also by epigenetic means^16^, can expand creating clones in the tissues^24,32^, such as in the case of CHIP^34^, which is associated with and possibly fueled by chronic inflammation^35^. Permanent DDR activation caused by irreparable lesions leads to cellular senescence establishment^66,67^ and may unleash genome instability when cell cycle checkpoints genes are inactivated^21,77^. This is the case when DNA damage occurs at telomeres, either because critically short upon extensive proliferation^65^, or because of the accumulation of irreparable lesions^66–69^. It is possible that when the senescence burden exceeds a threshold, senescent cells cannot be effectively cleared by the immune system and therefore accumulate^114^. In parallel, cellular senescence spreads in a paracrine fashion through the SASP, creating a chronic state of low-grade inflammation^102,110–112,114,126^. This pro-inflammatory microenvironment contributes to the decline in immune function that further impairs the recognition and removal of senescent cells^115^. SASP^111^ and, in general, chronic inflammation^69^ can also fuel this vicious cycle by causing paracrine DNA and telomeric DNA damage. Conversely, damaged telomeres activate inflammation also through cytosolic DNA fragment sensing mechanisms^64,77^. Finally, immunosenescence has been connected to epigenetic dysregulation^53,54^, and retrotransposon activation by epigenetic derepression in senescent cells triggers cGAS-STING-mediated inflammation^6,7^.

In conclusion, the ability to counteract tumorigenesis may be compromised when the immune function is altered during aging, but similar immune changes can also occur when DNA repair is impaired, telomeres are damaged, immune cells are senescent, or chronic inflammation is established. Overall, these processes are profoundly intertwined and cooperate at the organism level in determining increased cancer and age-related disease risk with age (Figure 4), for this reason, they should be studied in carefully-thought animal models.

## Considerations on animal models to study the impact of aging on cancer

Animal studies should be carefully designed to delve into the molecular mechanisms underlying the aging and cancer links and to test potential therapeutic strategies. In general, preclinical studies usually involve young animals: a survey among researchers indicated that the age range commonly used for rodent models clusters around 2-3 months, usually because of costs and supply reasons, and to conform with published data^159^. The Jackson Laboratory (JAX®) considers mice old when they are 18 months or older, which correspond to 60 years in humans. Although all the murine studies considered in this review to probe the impact of aging in cancer use the same age threshold, except progeroid models, it’s not unusual to read in literature aging studies conducted in mice younger than 18 months old, potentially leading to incorrect conclusions. The observation that the very same oncogenic insult induced at different ages can lead to distinct tumor types with different transcriptional programs^160^ is probably the best example of how impactful animal age can be. Testing therapies in young mice for tumors typically developing in older adults may also be misleading because of the different microenvironments and the absence of multimorbidity. Interestingly, aged microenvironment can display sex-specific differences in proliferation and stress response, determining the phenotypes of the emergent cancer^161^. Scientists should be aware of this and, when possible, adopt cancer animal models consistent with the age and sex at which tumors form and are treated in humans. Most importantly, the scientific community should find ways to make geriatric mice more commonly available and affordable. In a pan-cancer whole-genome study, the observation of age-related differences in mutational landscape and burden in human tumors^29^ may indicate that clinical trials too should include appropriate age ranges, although enrolling older patients can be complicated (Box 2).

Besides chronological age, modulating different aspects of aging, such as DNA repair or telomere status, is important to study tumorigenesis. Animal models, compared to humans, allow the alteration of these aging pathways to test causal relationships, but researchers should be aware of their limitations (Table 1).

Regarding the contribution of the immune system in cancer studies, comparing immunodeficient and immunocompetent animals can help dissect the role of immunity in cancer^151^. While injecting tumor cells into an immunocompromised mouse can be convenient when using human samples, it does not recapitulate a physiological response and microenvironment. Other strategies to study the impact of an aged immune system are transplanting aged bone marrow into young mice (heterochronic BMT)^153,162^ or engineering the immune system to resemble an old one^115^.

Finally, different experimental cancer models have pros and cons^148,163,164^. Syngeneic models in which murine tumoral cell lines are inoculated in wild-type mice have the advantage of having a complete immune response but cancer cell lines display restricted heterogeneity that might not give informative results when testing anti-cancer therapies^164,165^. Xenograft models generated by injecting immune-deficient mice with human cancer cell lines or primary tumor samples are useful to test treatments but have the important drawback of an incomplete immune response^164,165^. An improvement is represented by humanized models, myeloablated mice reconstituted with a human immune system^164^, although these models are complex to generate and irradiation will inevitably produce a non-physiological level of senescent cells and SASP. In both syngeneic and non-syngeneic models, the tumor injection strategy does not fully recapitulate tumor genetic heterogeneity, and implantation cannot mimic the tumor initiation step. These considerations, including the fact that younger mice have better angiogenic capacity^166^, might explain the paradoxical results of the growth advantage of syngeneic tumoral cell lines in young immunocompetent mice compared to old ones^162,166–169^. Indeed, cell lines that promote vascularization are unaffected by the age of the recipient, indicating that also the specific cell line selected matters^166^. An alternative model to study cancer initiation and progression is represented by chemical carcinogenesis^61^, but it often involves acute non-physiological exposure to carcinogenic compounds. Finally, genetically engineered mouse models with altered tumor-related genes develop spontaneous tumors and immune response, but frequently all their cells bear the same oncogenic mutation, conveniently accelerating tumorigenesis but making this model distant from human occurrence.

In summary, to date, there is no perfect preclinical cancer model that reproduces human pathology, heterogeneity, microenvironment, and immune surveillance: developing such experimental models remains the goal of many researchers.

## Conclusions

Aging has pleiotropic effects and multiple age-related diseases often accumulate in individuals leading to increased multimorbidity in the older population^170^, therefore acting on fundamental aging mechanisms rather than specific conditions produces a holistic advantage^114^. Cancer is one of such age-related diseases and, given the rise in the population’s average age, promoting healthy aging can decrease cancer incidence and mortality. Even in early-onset cancers, such as the increasingly frequent colorectal cancer^1^, aging itself seems unrelated but the role of aging-associated pathways, like cellular senescence and inflammation, cannot be excluded. Such approach has demonstrated its use in the booming number of clinical trials for cancer and age-related conditions that repurpose therapies targeting their reciprocal pathways (Box 3). These new therapeutic strategies are based on discoveries made using animal models, which represent the best tools to dissect in a complex system how aging pathways causally influence tumorigenesis and to test and translate anti-aging and anti-cancer therapies.

In the present review, we focused on some fundamental aging driver mechanisms, namely genomic mutations, DNA damage, telomere maintenance, cellular senescence, and immune function. However, there are other equally important mechanisms that we have not addressed, including stem cell functionality, epigenetic drift, loss of proteostasis, mechanical properties of the extracellular matrix, and others. Given the complexity of the interaction between aging and cancer, many aspects of this relationship are still to be discovered and characterized. Understanding basic biology in animal models can shed light on potential therapeutic strategies to promote human healthy aging.

## Figures and Tables

**Table 1:** Table illustrating some of the differences in genome stability, telomere biology, and immunity between mice and humans.

	*Mus musculus*	*Homo sapiens*
**Average lifespan (years)** ^172^	2	79
**Genomic base substitution rate per year** ^13^	796	47
**Genomic insertion-deletion rate per year** ^13^	158	2.5
**Phenotypes upon mutations in DNA repair genes** ^49^	Often mild: multiple mutations or generations needed	Often severe: single mutation or generation needed
**Average telomere length at young age (kb)** ^172^	50	15
**Telomere shortening rate (kb/year)** ^172^	7	0.07
**Somatic cell telomerase expression** ^88^	yes	no
**Phenotypes upon mutations in telomerase genes** ^70,92^	At late generations in homozygosis	In heterozygosis
**Immunosenescence** **phenotypes** ^145,156^	T cell receptor diversity severely restricted, immunological aging phenotypes before geriatric age	T cell receptor diversity reduced, immunological aging phenotypes in geriatric age

## Box 1 Telomeres as determinants of lifespan

Long-read Nanopore sequencing is a novel approach that recently allowed to show at unprecedented resolution that telomeres of human leukocytes are on average 6 kb long at young age and shorten around 30 bp/year^173^. Interestingly, mice have a much shorter lifespan but are born with much longer telomeres, which however shorten more than 100 folds faster^171,172^. Indeed, the mere correlation between telomere length and species longevity is controversial^88,174–177^. Rather, slower telomere shortening rate has been correlated with a longer lifespan in different species^172,178,179^, resulting in telomere erosion to the same proportion of the initial length at the end of life of different species^172^. Faster telomere shortening of short-living species indicates an inability to maintain telomere integrity through telomere elongation mechanisms and protect them from damage and erosion^68^. In line with this observation, human centenarians have superior telomere length maintenance abilities^147^. Most likely, both inherited telomere length at birth and shortening rates participate in determining aging and maximum lifespan; indeed, lifespan in mice correlates with the accumulation rate of short, thus dysfunctional, telomeres^171^. Intriguingly, mammals with different body sizes have evolved different strategies to maintain telomere length: species with larger body sizes generally have short telomeres and restricted telomerase expression, while smaller mammals have long telomeres and express telomerase more widely^88^. The formers have favored replicative aging as a tumor suppressor mechanism, while the latter possess a greater oxidative stress tolerance and replicative capacity thanks to long telomeres. This observation may contribute to address Peto’s paradox, whereby animals with more cells do not show higher cancer incidence than small animals^180^.

## Box 2 Aging in cancer clinical trials

Even though half of all tumors are diagnosed in people over 70 years old^4^, clinical trials are biased in favor of younger patients. Systematic reviews of clinical trials concluded that the percentage of enrolled patients older than 65 years is often lower than that expected based on cancer type at the year of enrollment^181^, and that the median age of participants is lower than the general population one, a gap which is widening over time^182^. Thus, the age of cancer patients at diagnosis and the one of subjects of clinical trials diverge, however this discrepancy is in part justified by the higher multimorbidity in the aged population^170^, which makes data interpretation and follow-up more complicated.

A review of phase 1 clinical trials reported a generally similar toxicity and therapeutic benefit in patients younger or older than 65 years^183^. Similarly, immunotherapies, such as immune checkpoint inhibitors, seem equally effective in younger and older adults^184^, albeit with some contradicting reports^145^. In addition to the paucity of clinical data in older cancer patients, these conclusions are biased by the important confounding factor that older people enrolled in clinical trials are often chosen as the healthiest, with the least comorbidities, thus not truly representing the aged population at large^145,184^. Indeed, phenotypical age or frailty level, also estimated using epigenetic clocks, may predict therapy response better than mere chronological age, highlighting the importance of considering them when performing clinical studies.

Between 2000 and 2015, oncology was the therapy area with the lowest drug approval rate, namely 3.4% of the drugs that entered phase 1 trials, with the bottleneck between phase 2 (the phase in which efficacy is tested) and 3^185^. This observation may indicate poor translatability in humans due to the low predictive power of assays and models used in preclinical studies^163^. Also for this reason, animal models should mimic human biology, including the changes associated with aging. Changes in genomic instability, telomere maintenance, cellular senescence, and immune function can all influence therapeutic effects during aging. For instance, genomic instability, including the one arising from telomere dysfunction, could provide the heterogeneity and plasticity needed for therapy resistance^186,187^. Moreover, senescent cells are non-proliferating, thus typically non-targeted by chemotherapy, which instead causes TIS and promotes inflammatory side effects^113,126,138^. Finally, the senescent immune system is skewed toward an immunosuppressive response, which can drive resistance to immunotherapy^188^.

In conclusion, further studies are needed to explore how the aged microenvironment can influence the response to anti-cancer treatments. If possible, careful consideration should be given when selecting age ranges for patients in clinical studies and when choosing animal models in preclinical studies.

## Box 3 Reciprocal repurposing of therapeutic strategies targeting aging and cancer

From an evolutionary perspective, aging is tightly linked to tumor suppressor mechanisms, thus approaches targeting age-related conditions need to avoid promoting cancer. Conversely, the need to target cancer without causing premature aging is equally important, since the most common cancer therapies, namely radio- and chemotherapy, can induce cellular senescence and frailty^56,138,139^. Despite this, the observation that many of the hallmarks of aging and cancer are shared points to the fact that therapeutic approaches that alleviate age-related symptoms may also reduce the risk of developing tumors, and vice versa. A possibility is to boost DNA repair by acting on the epigenetic regulators sirtuins^7,11^, which may improve lifespan and protect against carcinogenesis^17^. Interestingly, the four HDAC inhibitors currently approved for cancer treatments and those currently in clinical trials may act through different mechanisms, with senolysis being one of them^132^. Moreover, small molecules inhibiting LINE-1 retrotransposon endonuclease may be beneficial both in cancer and aging phenotypes since they decrease DNA damage and senescence-associated inflammatory markers^189^. Thanks to the understanding of telomere role in cancer, drugs targeting telomerase are presently under investigation for their anti-cancer potential^60^ and the first telomerase inhibitor has been recently FDA-approved for lower-risk myelodysplastic syndromes^100^. However, the observations that not all tumors rely on telomerase and that its inactivation may have opposite consequences on tumor development and age-related phenotypes indicate that therapeutic strategies that modulate telomerase activity should be carefully considered. A different and novel approach may consist of targeting shelterin components to make telomeres a vulnerability in cancer cells^190^. An additional example of the virtuous crosstalk between cancer and aging studies is the recent development of antisense oligonucleotides against telomeric transcripts, proposed as potential therapies for ALT cancers and progeric and telomere-driven age-related conditions^63,74–76^. Senotherapies are promising strategies in the anti-aging and anti-cancer fields, and the efficacy of some senolytics is currently evaluated in clinical trials for age-related conditions and frailty^11,114^, also in cancer survivors^56^. Notably, nutlin-3a, initially used in cancer research to stabilize p53, is now investigated as a senolytic in clinical trials for knee osteoarthritis^114^. CAR-T cells too, which are FDA-approved living drugs for hematological tumors, may have the potential to eliminate senescent cells^125,143^. Therapies that modulate epigenetic regulators are now undergoing clinical trials for cancer treatment, and there are indications they can act as senomorphics too by disrupting SASP^103^. In the last decade, immune checkpoint inhibitors have been used to enhance the effector anti-tumor response in different malignancies^148^, but they may have additional beneficial effects by boosting the immune response in older adults. Similarly, bone marrow transplant, which may rejuvenate the whole individual, is already a treatment for some blood cancer types and may also improve the anti-tumoral immune response^155^. Anti-inflammatory drugs, such as aspirin^11,145,152^, or inhibitors of specific cytokine pathways, such as anakinra, which is an interleukin-1 receptor antagonist already approved for immune disorders^155^, are being repurposed to fight tumor growth by rewiring the immune response and by reducing the risk for clonal expansion^35^. Many more interventions are possible and are not discussed in detail here, including lifestyle modifications such as diet and exercise. Since aging and cancer pathways are interconnected, these therapies have the great advantage of acting on multiple hallmarks simultaneously, illustrating the need to gain a deep understanding of how these pathways interact.
